# Endothelial Cells Promote Expansion of Long‐Term Engrafting Marrow Hematopoietic Stem and Progenitor Cells in Primates

**DOI:** 10.5966/sctm.2016-0240

**Published:** 2016-10-14

**Authors:** Jennifer L. Gori, Jason M. Butler, Balvir Kunar, Michael G. Poulos, Michael Ginsberg, Daniel J. Nolan, Zachary K. Norgaard, Jennifer E. Adair, Shahin Rafii, Hans‐Peter Kiem

**Affiliations:** ^1^ Clinical Research Division, Fred Hutchinson Cancer Research Center, Seattle, Washington, USA; ^2^ Howard Hughes Medical Institute, Chevy Chase, Maryland, USA; ^3^ Ansary Stem Cell Institute, Weill Cornell Medical College, New York, New York, USA; ^4^ Department of Genetic Medicine, Weill Cornell Medical College, New York, New York, USA; ^5^ Department of Surgery, Weill Cornell Medical College, New York, New York, USA; ^6^ Department of Physiology, Biophysics, and Systems Biology, Weill Cornell Medical College, New York, New York, USA; ^7^ Angiocrine Bioscience, New York, New York, USA; ^8^ Department of Medicine, University of Washington, Seattle, Washington, USA; ^9^ Department of Pathology, University of Washington, Seattle, Washington, USA

**Keywords:** Hematopoietic stem progenitor cell, Bone marrow transplantation, Endothelial cells, Gene therapy, CD34^+^ cells

## Abstract

Successful expansion of bone marrow (BM) hematopoietic stem and progenitor cells (HSPCs) would benefit many HSPC transplantation and gene therapy/editing applications. However, current expansion technologies have been limited by a loss of multipotency and self‐renewal properties ex vivo. We hypothesized that an ex vivo vascular niche would provide prohematopoietic signals to expand HSPCs while maintaining multipotency and self‐renewal. To test this hypothesis, BM autologous CD34^+^ cells were expanded in endothelial cell (EC) coculture and transplanted in nonhuman primates. CD34^+^C38^−^ HSPCs cocultured with ECs expanded up to 17‐fold, with a significant increase in hematopoietic colony‐forming activity compared with cells cultured with cytokines alone (colony‐forming unit‐granulocyte‐erythroid‐macrophage‐monocyte; *p* < .005). BM CD34^+^ cells that were transduced with green fluorescent protein lentivirus vector and expanded on ECs engrafted long term with multilineage polyclonal reconstitution. Gene marking was observed in granulocytes, lymphocytes, platelets, and erythrocytes. Whole transcriptome analysis indicated that EC coculture altered the expression profile of 75 genes in the BM CD34^+^ cells without impeding the long‐term engraftment potential. These findings show that an ex vivo vascular niche is an effective platform for expansion of adult BM HSPCs. Stem Cells Translational Medicine *2017;6:864–876*

**Significance Statement**

Transplantation of gene‐corrected bone marrow (BM) blood‐producing stem cells could be used to treat hematologic disease. Expansion of genetically corrected stem cells before reinfusion into the patient would improve engraftment, thus providing an effective therapy for genetic diseases. The results of the present study show that BM stem cells grown on endothelial cells engraft at high levels in the monkey. These results show that endothelial cells support stem cell expansion and are safe for use in transplantation studies and could thus be translated for use in the clinic for expansion of modified stem cells in gene therapy applications in patients.

## Introduction

Genetic diseases affecting the bone marrow (BM) and hematopoiesis can be treated by transplantation of gene‐corrected autologous or matched allogeneic hematopoietic stem and progenitor cells (HSPCs) or allogeneic HSPCs from umbilical cord blood (CB) donors. For standard of care, HSPCs are mobilized from BM and CD34^+^ cells collected from apheresis product [1]. Efficacy of gene‐modified HSPC transplantation (HSPCT) requires a threshold engraftment level, which is proportional to the CD34^+^ cell dose [1]. Many patients have predictive factors associated with poor mobilization [2, 3] and would thus be ineligible for HSPC gene therapy. Allogeneic HSPCT is an alternative to autologous HSPC gene therapy; however, identification of a human leukocyte antigen‐matched donor is often challenging. The side effects from allogeneic HSPCT can also be substantial and life‐threatening [4]. Therefore, development of a safe and effective BM HSPC expansion platform would increase the number of patients eligible for autologous HSPC gene therapy.

Several groups have developed human CB expansion platforms, including immobilized Notch ligand [5, 6], prostaglandin E_2_ (PGE_2_) [7, 8, 9], angiopoietins [10, 11], cytokines [12], and small molecules [13, 14]. In clinical trials, these were used for allogeneic CB, in which an expanded CB unit facilitated neutrophil recovery but might not engraft long term. To date, no expansion methods have been reported for clinical use with adult BM HSPCs.

We developed a vascular niche from human endothelial cells (ECs) that expands CB HSPCs with retained repopulation potential [15, 16]. The focus of the present study was to evaluate the vascular niche for BM HSPC expansion. We found that EC‐expanded gene‐modified BM HSPCs reconstituted long‐term hematopoiesis in the nonhuman primate. Our results indicate unprecedented marking in T lymphocytes, erythrocytes, and platelets, without selection. These findings reveal that EC‐mediated expansion of BM HSPCs is both safe and effective and, thus, has the potential to improve gene‐modified HSPCT for the treatment of lymphoid (HIV, severe combined immunodeficiency [SCID]) and erythroid (hemoglobinopathies) hematologic diseases.

## Materials and Methods

### CD34^+^ Cell Isolation and Transduction

Cell isolation, transduction, and transplantation for nonhuman primate controls were conducted as described previously [17, 18]. Marrow aspiration was performed, and CD34^+^ cells were isolated [19]. For ex vivo studies, CD34^+^ cells were cryopreserved and thawed directly onto ECs, as described. For in vivo studies with EC‐expanded gene‐modified cells and for unexpanded controls, CD34^+^ cells were collected from three naïve animals and transduced as described.

The CD34^+^ cell fraction was isolated from steady‐state bone marrow or primed bone marrow [17, 18] and plated into StemSpan‐Serum‐Free Expansion Medium (SFEM) (StemCell Technologies, Vancouver, BC, http://www.stemcell.com) with 100 ng/ml each of human stem cell factor (SCF), thrombopoietin (TPO), and FLT3 ligand (FL; all from PeproTech, Rocky Hill, NJ, http://www.peprotech.com). After overnight culture, the cells were transduced in media with 4 µg/ml protamine sulfate and 1 µg/ml cyclosporine (Cyclosporin A [Novartis, www.novartis.com]) in nontissue culture‐treated T‐75 flasks coated with 2 µg/cm^2^ RetroNectin reagent (CH‐296; Clontech, Mountain View, CA, http://www.clontech.com) at 10 × 10^6^ cells per milliliter in 10 ml.

For transduction, CD34^+^ cells were cultured overnight and transduced. The lentivirus vector SMPGW used in the present study has been previously described [18]. In brief, the concentrated lentivirus vector (4.5 × 10^8^ transduction units per milliliter) expresses human P140K‐MGMT, regulated by spleen focus‐forming virus promoter, and enhanced green fluorescent protein (GFP), regulated by human phosphoglycerate kinase promoter. Lentivirus vector (multiplicity of infection [MOI], 10) was added and cultured overnight. The second dose of lentivirus (MOI, 10) was added the next morning and transduced for an additional 8 hours (total transduction: MOI, 10 × 2). The animals underwent myeloablative irradiation (linear accelerator source, hyperfractionated over 2 days). Transduced cells were collected, washed, and infused into the irradiated animals. For ex vivo EC expansion with primed CD34^+^ cells, the cells were collected from 3 naïve donors as described previously [17, 18], cryopreserved, thawed, and plated onto ECs as described.

### CD34^+^ Cell EC Coculture, Preparation for Infusion, and Transplantation

E4ORF1^+^ human ECs were generated by Jason M. Butler [15, 20] or Angiocrine Bioscience (VeraVec ECs; Angiocrine Bioscience, New York, NY, http://www.angiocrinebioscienc.com). Two weeks before transplantation, ECs were thawed (3 million cells per flask) and passaged 1:2 (3 × 10^6^ cells per T‐75 flask). Gene‐modified BM CD34^+^ cells were thawed into StemSpan‐SFEM (1% P/S, 100 ng/ml SCF, TPO, FL; StemCell Technologies). Cocultures were fed every 3 days and passed 1:2 into fresh ECs if the cell concentration was >2 × 10^6^ cells per milliliter.

On the day of transplantation, the cells were resuspended in StemSpan SFEM containing 10 µM PGE_2_ (Cayman Chemical, Ann Arbor, MI, http://www.caymanchem.com), placed on ice, and vortexed every 30 minutes for 2 hours. The infusion product (autologous gene‐modified HSPC and human EC mixture) was filtered and suspended in 2% autoserum in saline. The animals underwent total body irradiation, 1,020 cGy, administered from a linear accelerator at 7 cGy/minute, as four equally divided doses 12 hours apart. Within 24 hours of radiation, cocultured HSPCs/ECs were intravenously infused into the donor animal. Hematopoietic recovery based on the complete blood count (platelet count >20,000 per microliter and absolute neutrophil count [ANC] >500) was defined as described previously [21].

### Real‐Time Quantitative Polymerase Chain Reaction Analysis

In vivo gene marking was analyzed by TaqMan 5′ nuclease real‐time quantitative polymerase chain reaction (PCR) assay as described previously [17]. Genomic DNA samples from primate white blood cells (WBCs) were analyzed in triplicate with a lentivirus‐specific primer/probe combination (forward, 5′‐TGAAAGCGAAAGGGAAACCA; reverse, 5′‐CCGTGCGCGCTTCAG; probe, 5′‐AGCTCTCTCGACGCAGGACTCGGC [Integrated DNA Technologies (IDT), www.idtdna.com]), with a GFP‐specific primer/probe combination (forward, 5′‐CTGCACCACCGGCAA‐3′; reverse, 5′‐GTAGCGGCTGAAGCACTG‐3′; probe, CCACCCTGACCTACGGCGTG) in a separate reaction with a β‐globin‐specific primer/probe combination (forward, 5′‐CCTATCAGAAAGTGGTGGCTGG; reverse, 5′‐TTGGACAGCAAGAAAGTGAGCTT; probe, 5′‐TGGCTAATGCCCTGGCCCACAAGTA [IDT]) to adjust for equal loading volume of genomic DNA per reaction.

### Hematopoietic Colony‐Forming Assays

Colony‐forming assays were performed [22]. Cells were plated into MethoCult 4230 (StemCell Technologies), supplemented with 100 ng/ml SCF, TPO, granulocyte colony‐stimulating factor (G‐CSF; Amgen, Thousand Oaks, CA, http://www.amgen.com), granulocyte macrophage colony‐stimulating factor, interleukin‐3 (IL‐3), IL‐6, and 4 U/ml erythropoietin (all from Peprotech), and scored 10 days later.

### Cytospin Analysis

A total of 100,000 cells were placed in cytocentrifuge, distributed onto slides, Wright‐stained, mounted, and analyzed.

### Flow Cytometry Analysis

The antibodies used were from BD Biosciences (San Jose, CA, http://www.bdbiosciences.com), unless indicated otherwise. WBCs were stained with antibodies: mouse anti‐human CD34‐PE (clone 563; catalog no. 550761), CD34‐APC (clone 563; catalog no. 561209), CD45‐APC (clone D058‐1283; catalog no. 561290), CD45‐V450 (clone D058‐1283; catalog no. 561291), and nonhuman primate‐specific CD38‐APC (Nonhuman Primate Reagent Resource, NIH, Bethesda, MD, http://www.nhpreagents.org). ECs and HSPC‐EC cocultures were also stained with KDR‐PE (catalog no. FAB357P; R&D Systems, Minneapolis, MN, http://www.rndsystems.com), CD31‐V450 (clone WM59; catalog no. 561653), CD31‐PE (clone WM59; catalog no. 555446), CD31‐APC (clone WM59; catalog no. 17‐0319; eBioscience Inc., San Diego, CA, http://www.ebioscience.com), CD144‐APC (VE‐cadherin; clone 16B1; catalog no. 17‐1449; eBioscience), and CD144‐Alexa Fluor 700 (clone 16B1; catalog no. 56‐1449; eBioscience), and Tra‐1‐85‐APC (catalog no. FAB3195A; R&D Systems). ECs were distinguished from HSPCs by forward and side scatter, expression of the pan‐human antigen CD147 (encoded by the human basigin gene), and identified by immunostaining with the Tra‐1‐85 antibody. For in vivo studies, WBCs were costained with CD13‐PE (clone L138; catalog no. 347837), CD14‐PeCy7 (clone M5E2; catalog no. 560919), CD3‐APC (clone SP34‐2; catalog no. 557597), CD20 BrilliantViolet605 (clone 2H7; catalog no. 302333; Biolegend, San Diego, CA, http://www.biolegend.com), CD4‐Alexa Fluor 700 (clone L200; catalog no. 560836), and CD8‐Pacific‐Blue (clone RPA‐T8; catalog no. 558207). For HSPC ontogeny analysis, CD34^+^ cells and EC cocultures were stained with the following antibodies: CD34‐PE, CD38‐APC, CD90‐PeCy7 (Thy1.1, clone 5E10; catalog no. 561558), CD45RA‐APC‐H7 (clone 5H9; catalog no. BDB561212), and CD49f‐PacificBlue (clone GoH3). Flow cytometry analysis was conducted on a BD LSR II flow cytometer machine (BD Bioscience). Fluorescence‐activated cell sorting (FACS) was conducted on a FACSAria II (BD Bioscience).

### Retroviral Integration Site Analysis

Mapping of integration sites (IS) was conducted on WBCs and sorted subsets, as described previously [22, 23]. Genomic DNA was extracted from leukocytes using the Qiagen blood DNA mini kit (Qiagen, Hilden, Germany, http://www.qiagen.com) in accordance with the manufacturer’s instructions. Lentivirus long terminal repeat (LTR)‐genome junctions were amplified by modified genomic sequencing‐PCR. Libraries were subjected to ion torrent semiconductor sequencing, and sequence reads were analyzed using custom Perl scripts (Dr. Grant Trobridge, Washington State University, Pullman, WA). Genomic sequences were mapped to the rhesus macaque genome (rheMac3) using a standalone version of Blat available from the UCSC Genome Browser (UCSC Genome Informatics Group, Center for Biomolecular Science & Engineering, Santa Cruz, CA, http://www.genome.ucsc.edu). Sequences corresponding to the same integration locus were grouped together to determine the total number of unique integration sites (clones) identified. The relative contributions of each clone were determined by the number of IS‐associated sequence reads corresponding to that clone. A quality control check was performed to reveal clones overrepresented by PCR bias by comparing the number of IS‐associated sequence reads with the number of different fragment lengths observed for each genomic locus.

### RNA‐Sequence Library Construction, Sequencing, and Analysis

RNA isolation, library construction, sequencing, and analysis were performed as described previously [24]. CD34^+^ cells from donors in the transplantation studies were subdivided into two subgroups. In group 1, unexpanded CD34^+^ cells went into TRIzol (Thermo Fisher Scientific Life Sciences, Waltham, MA, http://www.thermofisher.com) for RNA extraction. In group 2, CD34^+^ cells plated into EC coculture for 1 week, a fraction of which were FACS sorted to deplete the human ECs (by exclusion of human CD147^+^ cells by staining with Tra‐1‐85 antibody that does not cross‐react with monkey cells) and sorting on the CD34^+^ fraction (the cells were ∼98% CD34^+^ after 1 week of coculture). The second fractions were injected into nonhuman primates. All original RNA‐sequence data were uploaded to the NCBI Gene Expression Omnibus database (National Center for Biotechnology Information, NIH, Bethesda, MD, http://www.ncbi.nlm.nih.gov/geo/) with a public release date of September 1, 2015.

### Study Approval

Juvenile macaques (*Macaca nemestrina*) were housed at the University of Washington Regional Primate Research Center under conditions approved by the American Association for the Accreditation of Laboratory Animal Care. Healthy macaques were randomly assigned to the present study. The experiments were conducted under protocols approved by the institutional review board and the animal care and use committees of the University of Washington. Transplantation, priming (mobilization), and cell collection procedures and protocols have been previously described [17, 18].

## Results

### ECs Expand Macaque Primed and Steady‐State BM CD34^+^ Cells

We previously showed that human ECs support the expansion of mouse HSPCs [20] and human CB CD34^+^ cells [16] capable of hematopoietic reconstitution. We also showed that ECs support specification of hematopoietic progenitor cells from induced pluripotent stem cells through a Notch pathway mechanism [22]. In the present study, we hypothesized that a vascular niche would support expansion of BM HSPCs for reconstitution of nonhuman primates.

**Figure 1:**
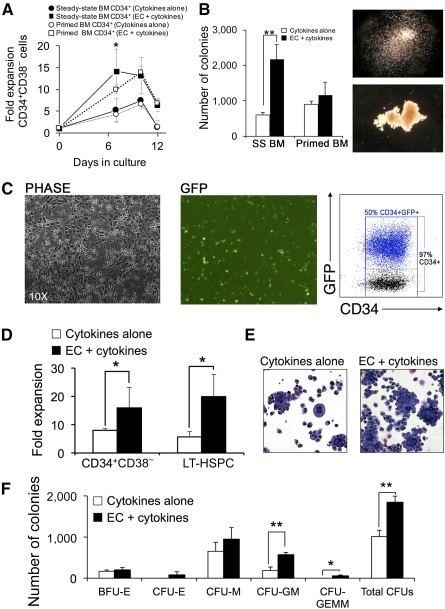
Human ECs support robust expansion of hematopoietic stem/progenitor cells. **(A):** Kinetics of expansion of SS and granulocyte colony‐stimulating factor/stem cell factor‐primed BM‐derived CD34^+^CD38^−^ cells after coculture with cytokines with or without human ECs (*n* = 3 naïve donors per condition). **(B):** Analysis of hematopoietic colony‐forming cell (CFC) potential of BM‐derived CD34^+^ cells after 7‐day expansion with cytokines with or without EC. Right: Images of CFU‐M (top) and BFU‐E (bottom) colonies. **(C):** Phase contrast (left) and fluorescent (middle) images of P140K‐MGMT‐GFP transduced macaque SS BM CD34^+^ cells and EC after 7 days of coculture. Right: Flow cytometry analysis of GFP and CD34 coexpression in gene‐modified CD34^+^ cells after EC expansion. **(D):** Summary of P140K‐MGMT‐GFP lentivirus‐transduced SS BM CD34^+^ cell expansion by flow cytometry analysis for detection of CD34^+^CD38^−^ and LT‐HSPC phenotype (CD34^+^CD49f^+^Thy1^+^CD38^−^CD45RA^−^; *n* = 3 naïve donors). **(E):** Microscopy showing morphology of Wright‐stained cytospin samples of gene‐modified CD34^+^ hematopoietic cells after coculture with cytokines with or without EC. **(F):** CFC analysis showing frequency and morphology of CFUs generated from gene‐modified CD34^+^ cells after expansion with cytokines with or without EC. Data are shown as the mean from the three experiments (donors) ± SD. CFC assays were conducted with three macaque donors and three biologic replicates per donor. Total CFUs are expressed per 10^5^ cells plated in MethoCult (StemCell Technologies). The colony types included BFU‐E, CFU‐M, CFU‐GM, and CFU‐GEMM. Statistical analysis used the Student *t* test: ∗, *p* < .05; ∗∗, *p* < .005. Abbreviations: BM, bone marrow; BFU‐E, burst‐forming unit‐erythroid; CFU, colony‐forming unit; CFU‐E, colony‐forming unit‐erythroid burst; CFU‐GEMM, colony‐forming unit‐granulocyte‐erythroid‐monocyte‐macrophage; CFU‐GM, colony‐forming unit‐granulocyte‐macrophage; CFU‐M, colony‐forming unit‐macrophage; EC, endothelial cell; GFP = green fluorescent protein; LT‐HSPC, long‐term hematopoietic and progenitor stem cell; SS, steady‐state.

To evaluate whether the vascular niche would support expansion of adult HSPCs, we compared the expansion of steady state BM CD34^+^ cells obtained from multiple naïve donors and primed/mobilized CD34^+^ cells collected from animals after treatment with G‐CSF/SCF as described previously [17, 18]. BM CD34^+^CD38^−^ cell expansion peaked on day 7, and expansion of primed CD34^+^CD38^−^ cells peaked on day 10 (Fig. 1A). Endothelial coculture supported 15‐fold expansion of steady‐state BM CD34^+^CD38^−^ cells, which was significantly higher compared with cells cultured in cytokines (10‐fold expansion) or primed CD34^+^ cells cultured with ECs (7‐fold expansion). Endothelial‐expanded BM CD34^+^ cells maintained higher colony‐forming potential compared with cytokine‐expanded BM CD34^+^ cells or EC‐expanded primed CD34^+^ cells (*p* < .005; unpaired two‐tailed *t* test; Fig. 1B).

To assess the suitability of the vascular niche for gene therapy, we evaluated EC‐mediated expansion of gene‐modified BM CD34^+^ cells. Marrow CD34^+^ cells from naïve donors were transduced with lentivirus vector expressing GFP and the chemotherapy‐resistant variant of the methylguanine methyltransferase gene (P140K‐MGMT), which may be used to expand gene‐modified HSPCs in vivo by treatment with alkylating chemotherapy, in the event of low gene‐modified cell engraftment [17]. CD34^+^/EC coculture contained 97% CD34^+^ cells, and 50% of these cells were GFP^+^ (Fig. 1C). The 7‐day EC coculture supported an ∼10‐fold increase in gene‐modified CD34^+^CD38^−^ cells and 17‐fold increase in long‐term (LT)‐HSPC‐like cells (CD34^+^CD90^+^CD49f^+^CD38^−^CD45RA^−^; *p* < .05, paired two‐tailed *t* test; Fig. 1D). Wright staining of cytospins from expanded HSPC samples revealed a greater number of blasts for EC‐expanded cells compared with cells expanded with cytokines alone (Fig. 1E). Colony‐forming cell (CFC) assays indicated that EC‐expanded cells gave rise to more mixed hematopoietic colonies (colony‐forming unit granulocyte macrophage [CFU‐GM], colony‐forming unit granulocyte‐erythroid‐macrophage‐monocyte [CFU‐GEMM]; paired two‐tailed *t* test, *p* < .005; Fig. 1F), which correlated with engraftment after HSPC transplantation [25, 26, 27].

### EC‐Expanded Gene‐Modified BM CD34^+^ Cells Engraft Without Toxicity

**Figure 2:**
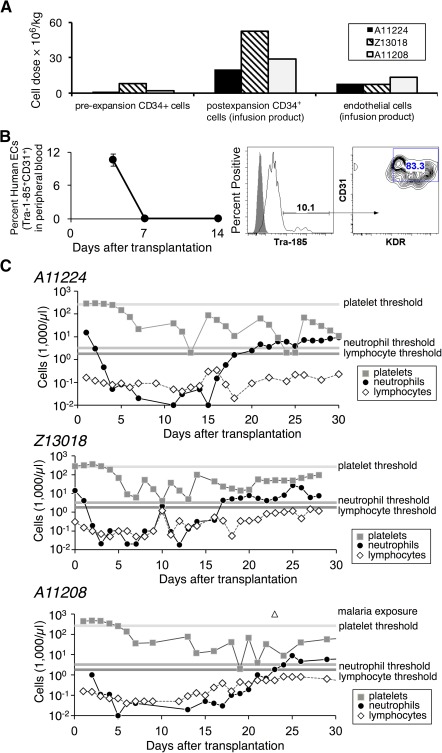
Hematopoietic reconstitution after transplantation with endothelial cell (EC) expanded CD34^+^ cells. **(A):** Cell doses used in autologous hematopoietic and progenitor stem cell transplantation in three nonhuman primates (animal identification nos.: A11224, Z13018, A11208). For each naïve animal, CD34^+^ cells were collected from bone marrow (BM), prestimulated with cytokines for 2 days, transduced with P140K‐green fluorescent protein lentivirus vector, cryopreserved, thawed, and then expanded in endothelial cell coculture for 7 days. Each animal received myeloablative conditioning (1,020 cGy) followed by intravenous infusion with a heterogeneous mixture of the transduced CD34^+^ cells and endothelial cell coculture. Cell doses are indicated per kilogram of body weight of BM‐derived transduced CD34^+^ cells before (pre‐expansion) and after (postexpansion infusion product) coculture with endothelial cells. The infusion products contained both the autologous CD34^+^ cells and the xenogeneic human EC dose. **(B):** Human ECs detected in peripheral blood up to 1 week after cell infusion. Data shown represent mean ± SD for *n* = 3 monkeys (A11224, Z13018, A11208). Representative flow cytometry analysis of peripheral blood sample 4 days after transplantation of CD34^+^/EC cocultures for detection of the human‐specific cell surface marker CD147 (Tra‐1‐85 antibody) and EC‐specific cell surface markers CD31 and KDR. **(C):** Hematopoietic recovery (over the first 30 days for each of the three indicated animals) as indicated by complete blood count analysis. Horizontal shaded lines represent minimal threshold of recover for (from top to bottom): platelets, neutrophils, lymphocytes. Abbreviation: EC, endothelial cell.

To determine the engraftment of EC‐expanded HSPCs, gene‐modified CD34^+^ cells/EC cocultures were transplanted into macaques (*n* = 3). EC coculture increased the CD34^+^CD38^−^ cell dose by 12‐fold. The mean CD34^+^ cell dose per kilogram was 35 × 10^6^ (range, 20–52 × 10^6^ CD34^+^ cells per kilogram; Fig. 2A). Infusion of high doses of EC‐expanded CD34^+^ cells did not cause any adverse events or hypersensitivity reactions. Coinfusion of the ECs did not cause infusional toxicity (i.e., vomiting, hypotension). ECs were detected in the blood up to 4 days after infusion but not thereafter (Fig. 2B).

Analysis of hematologic recovery revealed that the ANC was >500 cell per microliter by day 17 for two animals [28]. The third animal achieved neutrophil engraftment on day 21; this delayed reconstitution, blood transfusion requirements, and failure to recover platelet counts were attributed to latent malarial infection. For animals A11224 and Z13018, platelet recovery (>20,000 cells per microliter) without transfusion occurred after neutrophil recovery, consistent with the kinetics of recovery observed after clinical mobilized peripheral blood HSPCT (range, 16–20 days) [29]. These data show that ECs do not cause infusional toxicity and do not persist and indicate that EC‐expanded CD34^+^ cells exhibit similar engraftment kinetics to patients undergoing HSPCT.

### Long‐Term Multilineage Engraftment of EC‐Expanded Gene‐Modified CD34^+^ Cells

**Figure 3:**
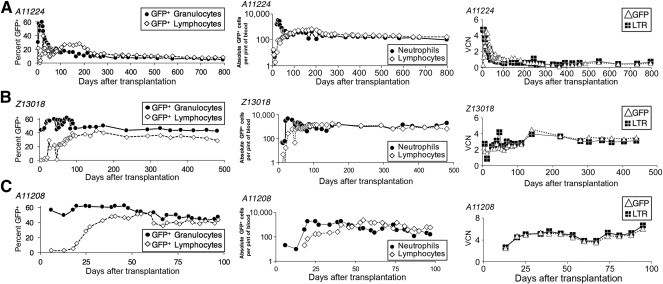
Long‐term engraftment and in vivo multilineage contribution to hematopoiesis by endothelial cell‐expanded gene‐modified autologous CD34^+^ cells. Detection of gene‐modified cells in animals A11224 **(A)**, Z13018 **(B)**, and A11208 **(C)**. Left: Percentages of GFP^+^ cells (gene marking by flow cytometry) in peripheral blood lymphocytes and granulocytes. Middle: Absolute cell numbers of GFP^+^ cells in peripheral blood lymphocytes and neutrophils. Right: Gene marking as determined by the mean VCN per circulating leukocyte genome equivalent (normalized to β‐globin copy number) by Taqman qPCR to detect the lentiviral LTR and GFP transgene. Abbreviations: GFP, green fluorescent protein; LTR, long terminal repeat; VCN, vector copy number.

**Figure 4:**
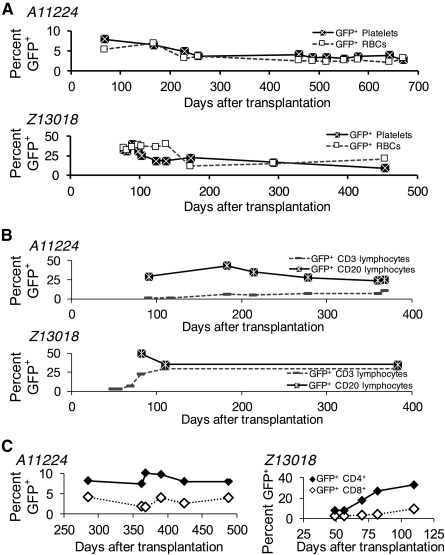
Detection of gene‐modified platelets, RBCs, and lymphoid subsets in vivo. **(A):** Detection of GFP^+^ platelets and RBCs in animal A11224 (top) and animal Z13018 (bottom). **(B):** Detection of GFP^+^ CD3^+^ T lymphocytes and CD20^+^ B lymphocytes in animal A11224 (top) and animal Z13018 (bottom). **(C):** Detection of GFP^+^ CD4 and CD8 T‐lymphocyte subsets in animal A11224 (left) and animal Z13018 (right). For determination of gene marking in lymphoid subsets, cells in the lymphoid subgate within the forward scatter by side scatter (FSC × SSC) gate were costained with fluorophore‐conjugated CD3, CD4, CD8, and CD20 antibodies. The percentages of GFP^+^ cells were then evaluated within the CD3, CD4, CD8, and CD20 subset gates. Abbreviations: GFP, green fluorescent protein; RBCs, red blood cells.

To confirm that EC‐expanded gene‐modified CD34^+^ cells contribute to hematopoiesis, transgene marking was analyzed in blood subsets after HSPCT. At the time of neutrophil engraftment, the percentage of GFP^+^ (gene‐modified) blood granulocytes was 50% in all animals (Fig. 3, left). By day +50, 25% of GFP^+^ lymphocytes were detected. GFP^+^ lymphocytes stabilized (A11224, Z13018) or doubled between days +50 and +100 (A11208). These data are consistent with HSPCT in historic monkeys and patients, in that the initial marking was higher in granulocytes than in lymphocytes, as myeloid reconstitution occurs before lymphocyte recovery [17]. The number of GFP^+^ granulocytes and lymphocytes correlated positively with the CD34^+^ cell dose and was 1‐log higher for the recipient that received the highest cell dose (Fig. 3, middle; Z13018, 60 × 10^6^ CD34^+^ cells per kilogram). Gene marking in lymphocytes >300 days after transplantation ranged from 11% to 34%, with up to 30% marking in CD3^+^ T lymphocytes (supplemental online Table 1). Marking in erythrocytes and platelets from A11224 and Z13018 were ∼5% and ∼25% GFP^+^ at days ∼700 and ∼450 after transplantation, respectively (Fig. 4A).

Analysis of marrow biopsies at day +120 revealed GFP marking in granulocytes (∼35%), monocytes (∼30%), and lymphocytes/blasts (∼20%), but no human ECs were detected (supplemental online Fig. 1). Six percent of LT‐HSPCs (CD34^+^CD38^−^CD90^+^CD49f^+^CD45RA^−^) in the marrow were GFP^+^. Gene marking in LT‐HSPCs at day +120 after transplantation was the same as the level of gene marking in the blood +800 days after transplantation (A11224). This finding is consistent with the canonical view of HSPC ontogeny, in which committed progenitors from HSPCs undergo proliferation on route to repopulation of the hematopoietic system with lineage‐committed mature blood cells [30].

We tracked engraftment by quantitative PCR analysis for detection of GFP and the lentivirus LTR, the latter to rule out contribution of lentiviral transduced (LTR^+^GFP^−^) ECs. No difference was found between the GFP and LTR viral copy number per circulating leukocyte (VCN), which stabilized at 2 and 5 VCN, respectively, for A11224, Z13018, and A11208 (Fig. 3, right).

### EC‐Expanded CD34^+^ Cells Reconstitute Polyclonal Hematopoiesis

**Figure 5:**
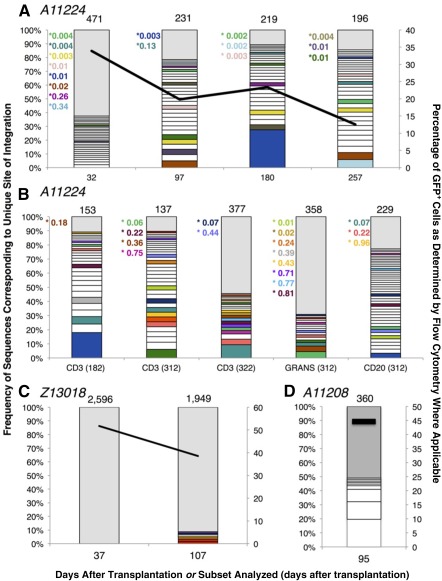
Gene‐modified peripheral blood leukocytes demonstrate polyclonal engraftment after endothelial cell (EC) expansion and myeloablative transplantation. Each graph represents the clonal diversity observed at each time point and/or in each blood cell subpopulation examined as a function of the frequency of genomic sequences identified in the pool (primary *y*‐axis) for each animal: A11224 **(A, B)**, Z13018 **(C)**, and A11208 **(D)**. Each bar designates the relative frequency of each clonal integration sequence identified in the sample from the most abundant (bottom of bar) to the least abundant (top of bar). The total number of unique sites of integration identified in each sample is listed at the top of each bar. Unique integration sites (clones) sequenced at a frequency >1% of the pool are designated by open boxes. All clones contributing <1% sequence frequency to the pool are grouped in a single gray box. Colored boxes designate clones tracked in multiple samples over time in the same animal. White boxes indicate the clones that were not observed in any other sample from the same animal. Clones tracked across multiple samples that did not contribute >1% frequency in a time point are designated by colored text citing the percentage of sequences identified within or next to the corresponding gray box. Overlay lines designate the percentage of GFP^+^ cells in the sample analyzed as determined by flow cytometry (secondary *y*‐axis). For **(B)**, the sorted lineage is indicated and the time point (days after transplantation) are indicated in parentheses. Abbreviation: GFP, green fluorescent protein.

To determine polyclonal repopulation, we performed retroviral integration site analysis on blood leukocytes. For the longest engrafted animal (A11224), reconstitution was polyclonal in bulk leukocytes. Two hundred different clones were detected at each time point (Fig. 5). We examined clonal diversity in sorted blood cell subsets. Between days +182 and +322, we observed no restriction of clonal diversity in CD3^+^ cells, and tracked multiple clones over time. At day 312, we detected shared clones in the CD3^+^, CD20^+^, and granulocyte lineages, demonstrating that EC‐expanded gene‐modified LT‐HSPCs were present at the time of infusion and were capable of sustaining hematopoiesis 1 year after transplantation. In Z13018, we also observed highly polyclonal reconstitution over time up to 107 days after transplantation, with nearly 2,000 individual clones identified at each time point (Fig. 5). Early reconstitution was so clonally diverse that not a single clone identified was found to contribute >1% of the genomes analyzed.

For A11208, we analyzed one time point (+95 days) and found polyclonal reconstitution. Based on the level of lentivirus gene‐modified blood leukocytes (44.6%), the overall contribution of the most abundant clone to leukocytes was ∼8.5%. This proviral integration mapped within the sequence encoding mRNA JV727540 in the rhesus genome (BGI CR_1.0/rheMac3 assembly). The function of this mRNA is unknown, and integration within this mRNA sequence has not previously been reported in conjunction with insertional mutagenesis; however, we cannot rule out the disease state in this animal (fatal malaria infection) as a possible cause for the clonal skewing observed at this time point. These data indicate that ECs promote polyclonal CD34^+^ cell expansion ex vivo.

### Whole Transcriptome Analysis Reveals Differential Gene Expression in Expanded and Unexpanded CD34^+^ Cells

**Figure 6:**
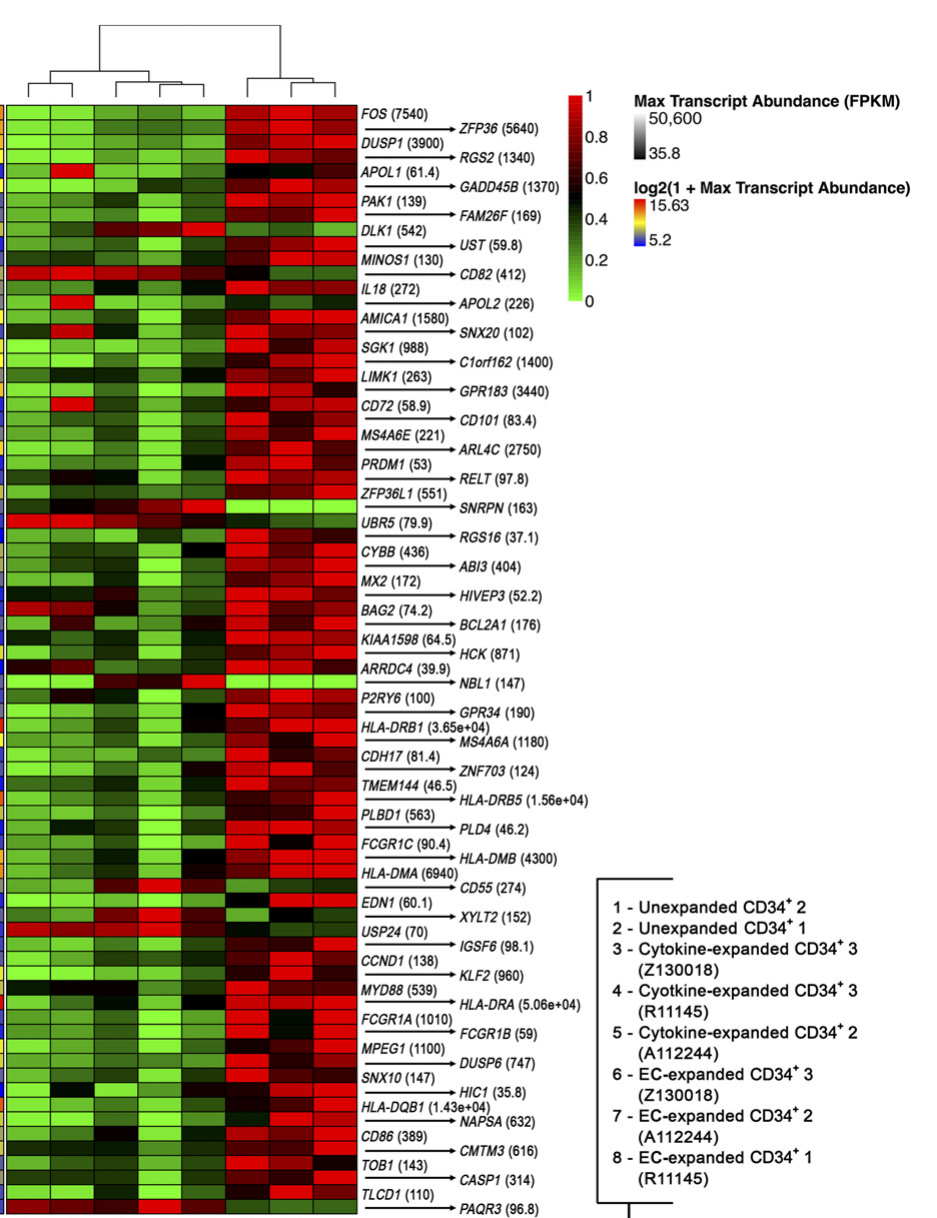
Differential gene expression in CD34^+^ cells unexpanded or expanded with cytokines or expanded with cytokines and ECs. Hierarchical clustering and transcriptional analysis of EC‐expanded CD34^+^ cells and cytokine‐expanded CD34^+^ cells from three donors (A11224, R11145, Z13018) and from unexpanded CD34^+^ cells (two donors). Heat maps display the relative abundance of genes that were differentially expressed across the three cell populations (unexpanded CD34^+^ cells, cytokine‐expanded CD34^+^ cells, and EC‐expanded CD34^+^ cells). EC‐expanded CD34^+^ cells were EC depleted and enriched for CD34^+^ cells before analysis. All other subsets were sorted according to expression of CD34 before analysis. Scaling is relative to the maximum FPKM for each gene across all samples. (For example, if the unexpanded CD34^+^ cells had the greatest mRNA abundance for a particular gene, all other values would be relative to the level in unexpanded CD34^+^ cells.) The numbers within parentheses next to each gene name provide that value of the maximum FPKM from all samples. All genes shown in this heat map are differentially expressed. The color scale to the left of the heat map is scaled to the FPKM values to the maximum. The genes are ordered according to the *p* value (from top to bottom, smallest to largest). For all genes shown, the following criterion was applied: FPKM cutoff of 20 (*p* ≤ .05, log twofold change). Abbreviations: ECs, endothelial cells; FPKM, fragments per kilobase of exon per million reads mapped; Max, maximum.

Given that EC‐expanded cells retain engraftment potential, we next compared the gene expression profiles of expanded and unexpanded CD34^+^ cells. CD34^+^ cells from three donors cultured on ECs for 7 days were FACS depleted of human ECs with Tra‐1‐85 antibody, which binds to the human CD147 surface antigen but does not bind to macaque cells [22], combined with immunomagnetic selection for CD34^+^ cells (Fig. 6). Transcriptome analysis of populations of CD34^+^ cells revealed that coculture with cytokines plus ECs or cytokines alone alters the HSPC transcriptional profile (Fig. 6). Of the 75 genes that were differentially expressed in EC‐expanded cells compared with the other populations, the most differentially expressed gene was *FOS*, which was identified as a putative downstream target of Notch signaling and also regulates hematopoietic stem cell (HSC) cycle entry and mitosis [31]. McKinney‐Freeman et al. found that highly proliferative fetal liver HSCs downregulate *FOS* [32]. Notably, *CD86* is also upregulated in EC‐expanded CD34^+^ cells. CD86^+^ HSCs have prolonged self‐renewal activity and lymphopoietic potential, and loss of these cells resulted in impaired lymphopoiesis in vivo [33]. These findings are consistent with previous studies indicating that the vascular niche balances HSPC self‐renewal and differentiation, in part, through Notch signaling [20]. We have shown that ECs expand repopulating CD34^+^ cells and alter their transcriptional profile without compromising engraftment, as indicated by the long‐term engraftment and robust lymphoid reconstitution achieved for animals A11224 and Z13018.

## Discussion

Methods that support ex vivo maintenance and/or expansion of long‐term repopulating bone marrow‐derived CD34^+^ cells would improve engraftment and therapeutic benefit for gene‐modified HSPC gene therapy applications. Validation of long‐term engraftment of expanded cells in a clinically predictive animal model would enable clinical trials and translation. We [15, 16], and other groups [34, 35, 36], have shown that human CD34^+^ CD38^−^ cells expand severalfold after coculture with endothelial cells and reconstitute hematopoiesis in vivo, with increased SCID mouse repopulation potential compared with either unexpanded or cytokine‐expanded CD34^+^ cells. In previously published studies describing EC‐mediated human BM CD34^+^ cell expansion from Chute et al., CD34^+^ CD38^−^ cells cocultured with primary ECs plus cytokines expanded 5‐fold [34] and 13‐fold [36]. In our studies, the EC and cytokine coculture system supported up to 17‐fold expansion of CD34^+^CD38^−^ cells (before PGE_2_ exposure).

In previously published studies of the nonhuman primate, expanded autologous BM CD34^+^ cells reconstituted hematopoiesis in and rescued lethally irradiated baboons after coculture with porcine microvascular endothelial cells (PMVECs) [37] or human brain microvascular endothelial cells [38]. These studies provided the first evidence that endothelial cells produce prohematopoietic factors capable of simultaneously increasing the CD34^+^ cell dose by severalfold while maintaining long‐term repopulating HSPCs. However, use of either xenogenic (porcine) or human brain ECs as an “off the shelf” HSPC expansion product is not clinically feasible, because sourcing ECs from xenogenic (pig) or adult human organs (brain tissue) for clinical use and expansion of primary unmodified ECs would present challenges.

Toward the clinical development of an HSPC expansion platform that could be an “off‐the‐shelf” product for use in clinical applications, we previously developed an ex vivo vascular niche from E4ORF1‐transduced, Akt‐activated endothelial cells from human cord blood, a source that is readily available and contains cells that are easily harvested [15, 16]. These Akt‐activated ECs produce additional prohematopoietic and angiocrine factors compared with unmodified primary ECs. In the present study, we demonstrated for the first time the ability of this ex vivo vascular niche to support CD34^+^ cell expansion, leading to long‐term engraftment of EC‐expanded BM CD34^+^ cells in a clinically relevant model. Transplant recipients showed long‐term (500–800 days after transplantation) multilineage polyclonal HSPC engraftment, robust gene‐modified lymphoid reconstitution, and gene marking in platelets and erythrocytes at levels not achievable previously after transplantation of mobilized/primed transduced CD34^+^ cells without in vivo selection. EC‐expanded CD34^+^ cells have a unique transcriptional profile, with upregulation of genes that are important for self‐renewal.

Although our data clearly demonstrate long‐term engraftment of EC‐expanded CD34^+^ cells, further studies focused on identifying the precise factors produced by ECs that maintain and expand LT‐HSPCs and/or enhance homing to and lodging in the marrow would facilitate clinical translation. Chute et al. [39], and others, demonstrated that transplantation of ECs alone rescues in vivo hematopoiesis of irradiated mice, highlighting the critical role of the vascular niche in hematopoietic regeneration. Regenerative hematopoiesis through contact with prohematopoietic factors is endothelial‐context dependent, as human HSPCs exposed to irradiation and then cultured with ECs recovered reconstitution potential while irradiated HSPCs cultured with cytokines alone did not rescue HSPC reconstitution [40]. Toward understanding the mechanism through which ECs support hemogenic regeneration after injury, subsequent studies in transgenic mice showed that conditional deletion of prohematopoietic factors in ECs, including vascular endothelial growth factor [41], SCF [42], chemokine C‐X‐C motif ligand 12 [43], Notch ligand Jagged‐1 [44], and pleiotrophin [45], impaired hematopoietic reconstitution. However, supplementing expansion cultures with prohematopoietic factors alone is not sufficient to maintain the HSPC reconstitution potential, suggesting that there are likely additional unidentified acellular paracrine or autocrine factors produced by the vascular niche that are required for LT‐HSPC maintenance.

Current CB HSPC expansion strategies have used small molecules [14] or BM mesenchymal cells [46] or have exploited the modulation of key signaling pathways [19] that balance HSPC homeostasis, self‐renewal, and differentiation. Csaszar et al. [47] demonstrated that Notch ligand delta‐1 (DL1)‐mediated HSPC expansion in the context of a fed‐batch system reduced IL‐6 cis‐ and trans‐signaling in CB HSPCs, increasing HSPC engraftment. Although such methods have been successful for expanding CB HSPCs, they have been almost exclusively evaluated with CB and have not translated to adult steady‐state BM. A few groups have applied these strategies to nonhuman primate mobilized CD34^+^ cells. Goessling et al. and others showed that mobilized CD34^+^ cells treated with 16,16 dimethyl PGE_2_ engrafted long‐term [7, 8, 9]. Although PGE_2_ increased homing, no difference was seen in the engraftment of PGE_2_‐treated mobilized CD34^+^ cells and untreated cells. In our study, gene‐modified BM CD34^+^ cells were expanded on ECs and then briefly pulsed with PGE_2_, which might have affected HSPC homing. However, the increase in CD34^+^CD38^−^ cell numbers that occurred was attributed to EC plus cytokine cocultures and not to PGE_2_, because the cells were not transduced, cultured, or expanded in the presence of PGE_2_. We hypothesized that the combination of EC expansion, EC coinfusion, and PGE_2_ pulse may synergize to improve CD34^+^ cell homing to, and lodging in, the bone marrow, thereby enhancing long‐term hematopoietic reconstitution.

Additional transplantation studies of gene‐modified CD34^+^ cells in nonhuman primates that evaluate the effect of each independent variable on HSPC engraftment would clarify the mechanism through which EC expansion and EC infusion independently increase hematopoietic reconstitution with BM CD34^+^ cells. Controlled studies in nonhuman primates (i.e., comparative engraftment of unexpanded CD34^+^ cells, unexpanded CD34^+^ cells coinfused with ECs, EC‐expanded CD34^+^ cells in which the EC fraction is depleted before infusion, cytokine‐expanded CD34^+^ cells alone, and unexpanded PGE_2_‐pulsed CD34^+^ cells) would reveal the effect that each independent variable has on hematopoietic reconstitution. Although we acknowledge that transient exposure to PGE_2_ could improve HSPC homing, acute treatment alone is likely not sufficient to rescue/recover the engraftment potential of an EC‐expanded CD34^+^ product that had lost all multipotent repopulating cells during ex vivo culture.

We have also tested other reagents that, like PGE_2_, showed promising results for CB CD34^+^ cell expansion and hematopoietic regeneration and reconstitution in mouse models. These methods included coculture of the Notch ligand DL1, mesenchymal stromal cells (MSCs), and forced expression of *HOXB4*. Watts et al. [48] expanded *HOXB4*‐transduced autologous nonhuman primate CB CD34^+^ cells, which had superior engraftment compared with unexpanded CB CD34^+^ cells. A follow‐up study to investigate whether activation of Notch signaling (through coculture with MSCs expressing Notch ligand DL) synergized with HOXB4 overexpression to further improve CB expansion and engraftment was tested in the autologous nonhuman primate setting [49]. That study showed that this combined expansion strategy accelerated neutrophil and platelet recovery compared with unexpanded and HOXB4‐expanded HSPC recipient historic controls. Although these methods, and others (PGE_2_, UM171, SR1), expanded CB CD34^+^ cells, no studies have been reported on the expansion of steady‐state BM CD34^+^ cells, until now.

Our findings showed that ECs support expansion of BM‐HSPCs (supplemental online Table 1 **)**, thus marking an important advance for HSPC gene therapy for patients who cannot tolerate mobilization but who would otherwise be candidates for gene therapy (e.g., sickle cell disease, myelofibrosis). Without mobilization, the yield from BM harvest results in a lower CD34^+^ cell dose. Because the success of CD34^+^ cell reconstitution is proportional to the cell dose, expansion of BM CD34^+^ cells would likely improve gene‐corrected HSPC reconstitution. ECs could thus be used to expand steady‐state BM for such patients.

In contrast to these expansion platforms, we provide the first evidence of a CB expansion strategy [16] that effectively translates to expansion of adult HSPCs to support hematopoietic reconstitution and long‐term engraftment of steady‐state adult BM CD34^+^ cells. We have shown for the first time that our ex vivo vascular niche expands steady‐state BM CD34^+^ cells while maintaining their stem cell properties (i.e., self‐renewal, multipotency, engraftment potential), leading to sustained long‐term engraftment (800 days), and enhances lymphoid and erythroid gene‐modified cell reconstitution. We have also shown that EC‐mediated expansion of HSPCs is safe, as the animals did not exhibit signs of infusional toxicity or any other health problems, such as unexpected thromboembolic or hemorrhagic complications that could be attributed to cotransplantation of ECs. As such, our studies set the stage for translation of endothelial cell‐mediated expansion of steady‐state BM CD34^+^ cells.

For the recipients of EC‐expanded CD34^+^ cells, hematopoietic recovery and engraftment occurred within the time frame observed in clinical BM HSPC transplantation (ANC >500 by days 14–20). Hematopoietic recovery and engraftment was achieved earlier compared with a previous study in which baboons were transplanted with similar BM CD34^+^ cell doses after expansion on PMVECs (ANC >500 by day 25 ± 1) [37]. The CD34^+^ cell dose per kilogram and the frequency of mixed hematopoietic colony‐forming units (CFU‐GM, CFU‐GEMM) have been shown to correlate with improved long‐term engraftment in patients [1, 25, 26, 27]. In our studies, the EC coculture supported up to 17‐fold expansion of CD34^+^CD38^−^ cells that substantially increased the CD34^+^ cell dose per kilogram with a concomitant increase in CFU‐GEMM. The expansion of CD34^+^ cells and CFCs achieved in the present study was substantially higher than the levels achieved in earlier reports evaluating EC‐ plus cytokine‐expanded CD34^+^ cells in baboons [37, 38]. However, similar to those previous reports, expansion of macaque CD34^+^ cells was lower compared with human CD34^+^ cell expansion with human cytokines and ECs [16], which can be attributed to the use of human ECs and human cytokines, as nonhuman primate reagents were not available. In addition to the increased cell dose and positive safety profile associated with vascular niche‐expanded marrow CD34^+^ cells, we also achieved unprecedented levels of gene marking in CD3^+^CD4^+^ T lymphocytes, erythrocytes, and platelets without the requirement for in vivo selection.

Our previous studies in the nonhuman primate indicated that myeloid cells typically have the highest efficiency of gene marking in vivo after HSPC transplantation. In those studies, achieving high levels of transgene expression in lymphoid, erythroid, and megakaryocyte progeny was more challenging despite polyclonal repopulation [17, 18]. In addition, gene marking in the lymphocyte pool was predominantly in B cells, with <20% in GFP^+^ CD20^+^ B lymphocytes [17]. In contrast, marking in CD3^+^ T lymphocytes was <5% [50] without in vivo selection. In the study by Trobridge et al. [50], in vivo selection with O^6^‐benzylguanine combined with alkylating chemotherapy (temozolomide) increased marking in CD3^+^ cells to 7%. In contrast, here we show that reconstitution of nonhuman primates with endothelial cell‐expanded BM CD34^+^ cells led to an unprecedented 30% gene marking in CD3^+^CD4^+^ lymphocytes (range, 10%–30%) without in vivo selection. This high level marking in gene‐modified T cells would likely improve the efficacy of HSPC and gene therapies for the treatment of HIV and genetic immunodeficiencies.

The level of marking achieved in erythrocytes and platelets after transplantation of endothelial cell‐expanded HSPCs is 2‐ to 10‐fold greater than the level previously reported by our group and others for erythroid‐specific marking after transplantation with lentivirus transduced mobilized HSPC transplantation [51, 52]. Therefore, the gene marking observed in the erythroid and megakaryocyte progeny of endothelial cell‐expanded CD34^+^ cell progeny in vivo is unprecedented, as neither mobilization before transplantation nor in vivo selection with chemotherapy after transplantation were required to achieve these milestones in our study.

In summary, vascular niche‐mediated expansion of steady‐state BM CD34^+^ cells has the potential to increase the accessibility and improve the outcome of success of HSPC gene therapy for patient populations with varied hematopoietic disease indications, from frequent immunodeficiencies (infectious or genetic), hemoglobinopathies (sickle cell disease) and anemias (Fanconi anemia) to rare orphan diseases.

## Conclusion

An ex vivo vascular niche sustains and/or expands bone marrow‐derived LT‐HSPCs that retain multilineage blood reconstitution of the clinically relevant nonhuman primate. These findings demonstrate safety and effectiveness of a bone marrow HSPC expansion platform that could be clinically translated to HSPC gene therapy applications.

## Author Contributions

J.L.G.: conception and design, performance of all experiments with technical support where indicated, manuscript writing and editing, final approval of manuscript; J.M.B.: generation of ECs, contribution to experimental design, manuscript editing, final approval of manuscript; B.K. and Z.K.N.: performance of RNA sequencing analysis and related bioinformatics, final approval of manuscript; M.G.P.: isolation of RNA and performance of RNA sequencing experiments, final approval of manuscript; M.G. and D.J.N.: generation of ECs, feedback on experiments, manuscript editing, final approval of manuscript; J.E.A.: performance of RIS analysis and related bioinformatics, final approval of manuscript; S.R. and H.‐P.K.: general guidance, manuscript editing, final approval of manuscript.

## Disclosure of Potential Conflicts of Interest

M.G. is an employee of Angiocrine Bioscience. D.J.N. is an employee of, and equity holder in, Angiocrine Bioscience. S.R. is a founder of Angiocrine Bioscience and an unpaid consultant. The other authors indicated no potential conflicts of interest.

## Supporting information

Supporting Information
