# Supplementary material for: Endothelial Cells Promote Expansion of Long‐Term Engrafting Marrow Hematopoietic Stem and Progenitor Cells in Primates
Source: Stem Cells Transl Med. 2016 Oct 14;6(3):864–76. doi: 10.5966/sctm.2016-0240 (PMC5442761; doi:10.5966/sctm.2016-0240)
Supplement: Supplementary file 1 — Supporting Information [file SCT3-6-0864-s001.pdf]

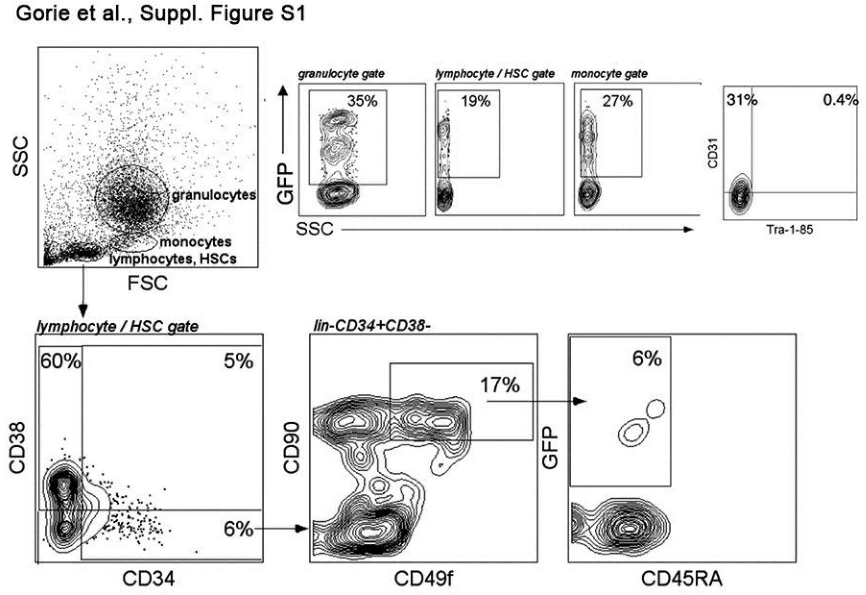

Supplemental Figure S1. Detection of GFP$^+$ cells in the bone marrow 3 months after transplantation of EC-expanded lentivirus transduced CD34$^+$ cells (Animal A11224). Detection of GFP$^+$ cells by flow cytometry in granulocyte, monocyte and lymphocytes gates (based on FSC X SSC). *Far left*: Absence of human (Tra-185$^+$) CD31$^+$ endothelial cells in the marrow. *Bottom:* Flow cytometry analysis of gene marking LT-HSPC like cells (CD34$^+$CD90$^+$CD49f$^+$CD38$^-$CD45RA$^-$).

Supplemental Table S1. Gene transfer, cell dose, and marking *in vivo* >300 days* after transplantation of EC-expanded $CD34^+$ cells. $\%FL^+$: % fluorophore positive.

Transplant details		***ex vivo analysis***		***in vivo analysis***			
**NHP ID**	**Marrow source**	**$CD34^+$ cell dose×10^6/kg**	**$\%FL^+$ $CD34^+$ cells**	**$\%FL^+$ Granulocytes**	**$\%FL^+$ Lymphocytes**	**$\%FL^+$ of $CD3^+$**	**$\%FL^+$ of $CD20^+$**
A11224	steady state (unprimed)	20	55	11	11	7	24
A11208	steady state (unprimed)	30	60	17	27	28	39
Z13018	steady state (unprimed)	60	52	43	34	30	35

After transplantation, all animals were treated with post transplantation immunosuppression for 180 days to prevent immune response to GFP (foreign protein). *Analysis for A11208 was conducted at time of death due to malarial infection (100 days' post transplantation).
